# *In vivo* therapeutic effect of combination treatment with metformin and *Scutellaria baicalensis* on maintaining bile acid homeostasis

**DOI:** 10.1371/journal.pone.0182467

**Published:** 2017-09-06

**Authors:** Kyungsun Han, Shambhunath Bose, Jing-Hua Wang, Soo-kyoung Lim, Young-Won Chin, Young-Mi Kim, Han-seok Choi, Hojun Kim

**Affiliations:** 1 Department of Rehabilitation Medicine of Korean Medicine, Dongguk University, Goyang, Gyeonggi-do, Republic of Korea; 2 Clinical Research Division, Korea Institute of Oriental Medicine, Daejeon, Republic of Korea; 3 Applied Surface Technology Inc., 11th Floor, Bldg. A, Advance Institutes of Convergence Technology, Suwon, Republic of Korea; 4 College of Pharmacy, Dongguk University, Goyang, Gyeonggi-do, Republic of Korea; 5 Department of endocrinology, Dongguk University, Goyang, Gyeonggi-do, Republic of Korea; Beckman Research Institute, UNITED STATES

## Abstract

The radix of *Scutellaria baicalensis* (SB) is a herb widely used in traditional Chinese medicine to treat metabolic diseases. Several main components, including baicalin and wogonoside, possess anti-dyslipidemia, anti-obesity and anti-diabetic effects. We hypothesized that co-administration of SB extract and metformin exerts a better effect on obesity-induced insulin resistance and lipid metabolism than treatment with metformin alone. We compared the effect of metformin (100 mg/10 mL/kg/day) alone with co-administration of metformin (100 mg/5 mL/kg/day) and SB extract (200 mg/5 mL/kg/day) on Otsuka Long Evans Tokushima Fatty rats, a useful model of type II diabetes with obesity, and used Long-Evans Tokushima Otsuka rats as a control. Weight, fasting glucose, oral glucose tolerance test, intraperitoneal insulin tolerance test, and serum total cholesterol were measured after 12 weeks of drug administration. We observed a synergetic effect of metformin and SB on lowering cholesterol level by excretion of bile acid through feces. We found that this accompanied activation of *FXR*, *CYP7A1* and *LDLR* genes and repression of *HMGCR* in the liver. Although there were no significant changes in BSH-active gut microbiota due to high variability, functional prediction with 16S sequences showed increased primary and secondary bile acid biosynthesis in the combination treatment group. Further study is needed to find the specific strains of bacteria which contribute to FXR-related cholesterol and bile acid regulations.

## Introduction

Metformin, a first-line medication for type II diabetes mellitus, stimulates AMP-activated protein kinase (AMPK) activity in the liver, decreasing hepatic glucose production while increasing glucose utilization in skeletal muscle. However, a systematic review concluded that metformin has no effect on blood pressure or dyslipidemia in type 2 diabetes patients [1]. There are efforts to find effective cocktail drugs with metformin to give a broader treatment opportunity for metabolic diseases. Reasonable candidates for the combination treatment with metformin are herbs traditionally used for treating metabolic diseases.

The root of *Scutellaria baicalensis* (SB), also known as *Scutellariae radix* or skullcap root, is widely used in traditional Oriental medicine for its beneficial effects such as antioxidant [2], anti-tumor [3], anti-inflammatory [4, 5], antiviral [6], neuroprotective [7] and anti-diabetic effects [8]. Abundant flavonoids in SB are responsible for these pharmacological effects. Although the principal constituents of SB are baicalin, baicalein and wogonin [9], SB contains almost 70 polyphenols such as chalones, flavanonols, and anthocyanidines [10–12]. In this study, we hypothesized that co-administration of SB extract and metformin could exert a better effect on obesity-induced insulin resistance and lipid metabolism than treating with metformin alone.

When SB ethanol extract and metformin were co-administered to streptozotocin-induced diabetic rats, hepatic antioxidant enzymes such as superoxide dismutase and catalase were elevated [8]. Plasma and hepatic triglycerides and cholesterol levels were significantly reduced while pancreatic insulin increased [8]. However, little is known about underlying mechanisms of such beneficial effects. We used PCR array analysis to screen for genes related to fatty liver and insulin resistance after combination treatment with metformin and SB. Beyond the anti-diabetic effect of metformin, we found a possible synergetic effect of meformin and SB on maintaining bile acid excretion and contributing to lowering plasma triglyceride. Growing evidence suggests intestinal microbiota is important in development of metabolic syndrome [13]. The intestinal microbiota is critical in transforming primary bile acids to secondary bile acids so that the chemical diversity of bile acid increases [14, 15]. Intestinal microbiota also interact with drug responses [16]. Metformin, for example, influences short-chain fatty acid production, regulates gut hormone and bile acids, thus influencing gut microbial composition and human metabolism [17, 18]. Considering the extensive interaction between host metabolism, intestinal microbiota and drug administration, we analyzed representative microbiota by 16S rDNA bacterial pyrosequencing analysis.

Our results suggest that co-administration of metformin and SB facilitates cholesterol to bile acid conversion and promotes fecal loss of bile acid and cholesterol through FXR-related pathway. Moreover, we propose a possible synergetic effect involving intestinal microbial change when metformin is co-administered with SB.

## Materials and methods

### Preparation of Scutellaria baicalensis extract and metformin

**Fig 1:**
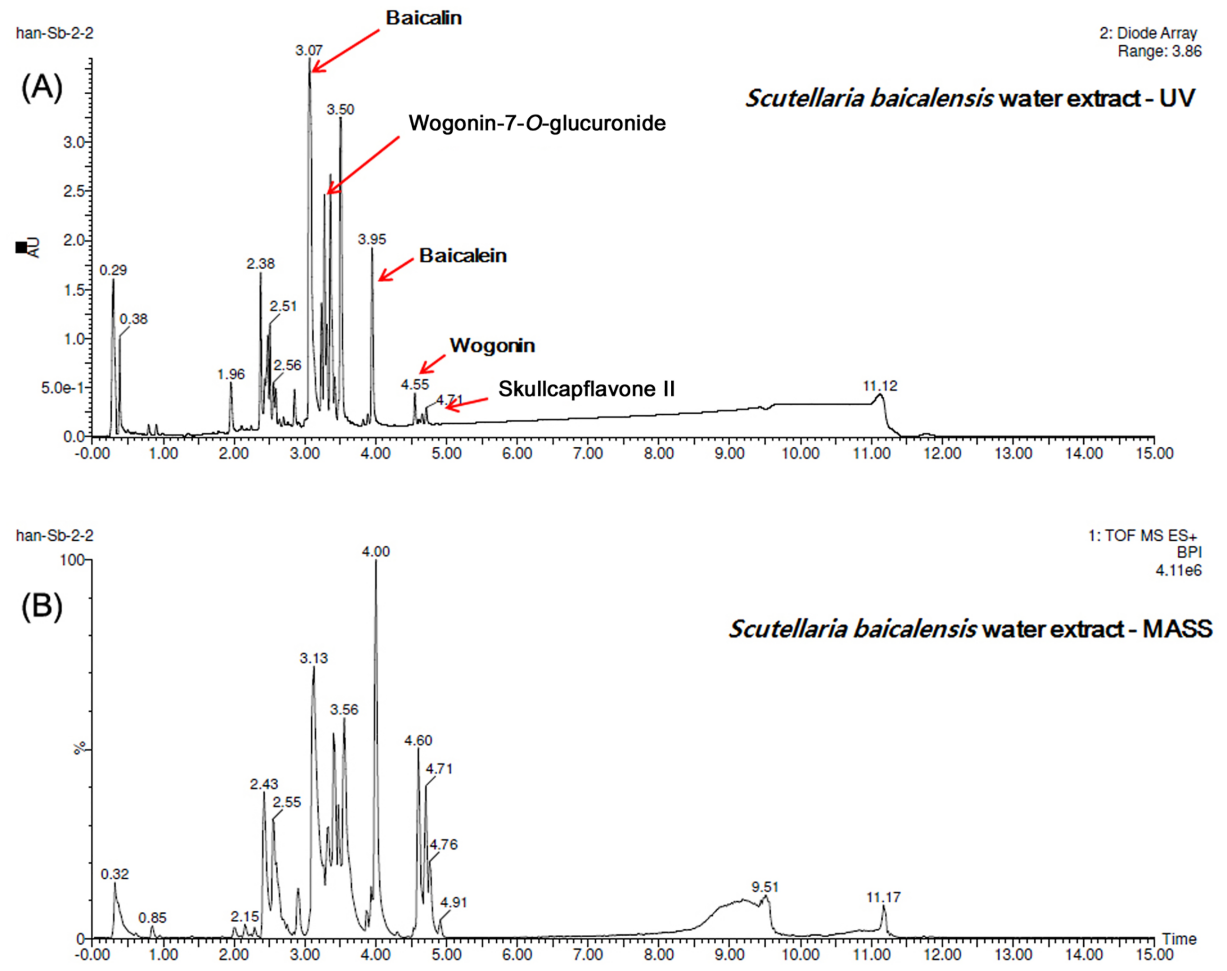
Quality control of Scutellaria baicalensis(SB). Detection of main components of SB extract by Ultra-Performance Liquid Chromatography (UPLC) analysis. Main compounds were confirmed by comparison of the retention time, UV chromatogram(A) and MASS chromatogram(B) with those of the standards. The major compound was identified as baicalin.

The dry root of *Scutellaria baicalensis*(SB) was supplied by Dongguk University Ilsan Hospital. After thorough washing, 100g of powdered SB was extracted in 300L of boiled water for 3 hours. The extraction was filtered with 8ym pore size qualitative filter paper, and the extract was freeze-dried.SB extract was analyzed with a Waters Acquity^™^ Ultra Performance LC system (Waters Corp., Milford, MA) equipped with a ACQUITY UPLC^®^BEH C18 column (2.1 mm×50mm, 1.7 μm, UK, temperature of 40°C). The mobile phase A was 0.1% formic acid in Water and mobile phase B was0.1% formic acid in Acetonitrilie. They were mixed with gradients for the analysis as flowing: 10% B during 1 min, from 10% to 100% in 10 min, 100%in 10-12min., 100 to 10% in 12–12.5min and 10% in 12.5-15min. Run time was 15 min, the mobile phase flow rate was: 0.6ml/min and UV detection was performed at 190–500 nm. Baicalin was the main component of SB (Fig 1).

### Animals and experimental schedule

The animal study was approved by the Institutional Animal Care and Use Committee (IACUC-2014-037) in Dongguk University and was performed in accordance with the Guide for the Care and Use of Laboratory Animals (Institute of Laboratory Animal Resources, Commission on Life Sciences, National Research Council, USA; National Academy Press: Washington D.C., 1996). Seven four-week-old male LETO rats and 21 OLETF rats were purchased from Otsuka Pharmaceutical Co. (Tokushima, Japan). They were housed in a specific pathogen free facility under conditions of controlled temperature (20 ± 2°C), relative humidity (40%-60%), with a 12-h light-dark cycle (lights on at 7:00 a.m.). The animals were given access to water and a standard normal chow diet (Soyagreentec, Hwaseong-Si, Korea) containing 20% protein, 4.5% fat, and 63% calories from carbohydrate *ad libitum*. After six weeks of acclimatization, seven of the OLETF rats were orally treated with metformin (100 mg/10 mL/kg/day) for 12 weeks, seven OLETF rats were orally treated with combination of metformin (100 mg/5 mL/kg/day) and SB (200 mg/5 mL/kg/day) for 12 weeks, and the remaining seven OLETF and LETO rats were orally administrated distilled water using the same volume and frequency as other groups. Based on clinical dosage (8-10g/day/60kg for SB and 500-2500mg/day/60kg for Metformin), we estimated the dosage of the experiment. We calculated human equivalent doses based on body surface area considering 10% extract yield of SB [19].

Body weight and food-intake was measured once a week. Fresh stool samples were collected the day before sacrifice and stored at -80°C immediately. After 12 weeks dosing and 12 hours fasting, all of the animals were sacrificed. Blood samples were drawn from the ventral aorta and rapidly transferred into a BD Vacutainer (Franklin Lakes, NJ, USA). After two hours of blood clotting, serum was separated by centrifugation under 3,000 × g for 15 min at room temperature. Liver and fat tissues were removed, weighed, and rapidly stored in liquid nitrogen for future analysis. One animal in OLETF group died during the experiment for unknown reason. Thus, all data for the OLETF group includes only six animals.

### Oral glucose tolerance test (OGTT) and intraperitoneal insulin tolerance test (IPITT)

The third day before the end of the animal experiment, 12 hours fasting adapted rats were treated with sterilized glucose solution (2 g/kg, Sigma, USA) by oral gavage. The blood glucose values were measured using ACCU-CHEK Active (Roche Diagnostics, Germany) via the tail needle-punched blood drops at 0, 30, 60, 90, 120 min after glucose treatment. The second day before the end of animal experiment, 12 hours fasting-adapted rats were administrated biosynthetic human insulin (0.75 U/kg, Eli Lilly and Company, IN, USA) by intraperitoneal injection. Blood glucose values were determined by ACCU-CHEK Active (Roche Diagnostics) via the tail needle-punched blood drops at 0, 30, 60, 90, 120 min after insulin injection. Both of the OGTT and IPITT results were expressed as areas under the curves (AUC) to evaluate the degree of the glucose tolerance impairment and insulin sensitivity separately.

### Serum biochemical analyses

Serum triglyceride (TG) and total cholesterol (TC) level were determined using commercial enzymatic assay kits according to the manufacturer's instructions (Asan Pharmaceutical Co., Seoul, Korea). Serum insulin was measured by a Rat Insulin ELISA Kit (Mercodia, Sweden). Briefly, 10 μL of samples or standards combined with 100 μL of enzyme conjugate solution were added to a pre-coated plate, followed by incubation for 2 hours at room temperature. After washing six times with 700 μL/well wash buffer solution, 200μL substrate TMB was added, followed by incubation for 15 min at room temperature. Finally, 50μL stop solution was added and the plate was read immediately on a spectrophotometer at 450 nm. The concentration of fasting serum insulin was calculated by standard curve.

### RT^2^ Profiler PCR array

Liver tissues were homogenized in lysis buffer and total RNA was isloated with RNeasy Mini kit (Qiagen, Valencia, USA) according to the manufacturer's instructions. First strand complementary DNA was synthesized using RT^2^ first strand kit (Qiagen) with 200 nanograms of total RNAs. RT Profiler PCR array rat fatty liver (PARN-157Z, Qiagen) is used to analyze 84 gene sets of interest. For group comparison, we used cDNA from four randomly selected rats from each group pooled together. All PCR experiments were conducted with StepOne Real-Time PCR Systems. Software supplied by the instrument manufacturer (Applied Biosystems, USA) was used for processing and analysis of the data.

### Western blot analysis

Liver tissues (20mg) were homogenized in 600μl protein extraction solution (iNtRON Biotechnology, Inc., Korea), with addition of protease inhibitor (Sigma, USA) and phosphatase inhibitor cocktail (GenDEPOT, USA). For Western blot analysis, 40μg of protein was fractionated by SDS-PAGE, transferred onto a PVDF membrane (Amersham, Japan). Membranes were blocked with 5% skim milk in Tris-buffered saline and probed with target primary and secondary antibodies. Total antibodies FXR (Cell signaling, USA), LDLR (Cell signaling, USA), CYP7A1(Cell signaling, USA), HMGCR (Millipore/Upstat, USA) and β-actin (Santa Cruz Biotechnology, USA) were used. Protein amount was expressed relative to the amount of β-actin Due to poor sample quality, all the proteins from each group were pooled together for the analysis.

### Quantitative analysis of total cholesterol in stool and liver

Frozen liver tissue (200mg) was homogenized in 1ml distilled water. Fecal samples were dried with a freeze dryer, and dried fecal matter (100mg) was homogenized in 900 μl distilled water. The homogenate was extracted by 5ml chloroform-methanol (2:1) mixture and centrifuged at 7,000 rpm for 5 min. The chloroform layer was aspirated carefully, dried and resolved by isopropanol. Liver TG and total cholesterol were determined using commercial enzymatic assay kits.

### Total bile acid concentration analysis

Quantification of total bile acids concentration from liver and fecal samples were conducted with Rat Total Bile Acids Kit (Crystal Chem, IL, USA) according to the manufacturer’s instruction. Liver tissues were homogenized in 1ml of 75% ethanol and incubated for 2 hours at 50°C. After centrifuging for 10min at 6000xg, 20ul of supernatant fractions and 150ul of reconstituted reagent were loaded into a microplate for determination of absorbance.

### Analysis of intestinal bacterial community

To describe the bacterial community, fecal samples collected at the beginning and end of the study in sterile containers which were brought to the laboratory, frozen, and stored at −80°C until analyzed. Bacterial DNA was isolated from stool samples using the FastDNA SPIN Kit (MP Biomedicals, Santa Ana, CA). 16S rRNA regions were amplified with 30uL of PCR premix solution containing 30ng of purified DNA as a template, 1.25U Ex Taq DNA polymerase, 5 uL 10X Ex Taq buffer, 3mM MgCl2 and 0.2mM dNTP mix (SolGent, Daejeon, Korea). The V1 and V2 hyper-variable regions of bacterial 16S rRNA gene were amplified with TOP simpleTM DryMIX solution (Enzynomics, Daejeon, Korea) by the primer pair 8F (5’-AGAGTTTGATCCTGGCTCAG-3’ and 338R (5’-TGCTGCCTCCCGTAGGAGT-3’) containing 8-base sample-specific barcoded sequences and common linker (TC for forward and CA for reverse primer) sequences at the 5’end. Thermocycling was conducted in a C1000 Thermal Cycler (Bio-Rad, California, USA) under the following conditions: initial denaturation at 94°C for 2 min; 30 cycles of denaturation at 94°C for 30s, annealing at 55°C for 30s, and extension at 72°C for 1min; and a final extension at 72°C for 10min. Amplified samples were purified with the QIAquick PCR Purification kit (Qiagen). PCR amplicons (100ng) tagged with the sample-specific barcode sequences were pooled. The DNA quantity and quality were further assessed on a BioAnalyzer 2100 microfluidics device (Agilent, California, USA) with a DNA1000 lab chip (Agilent). Pooled DNA was amplified by emulsion polymerase chain reaction using 454 pyrosequencing Genome Sequencer FLX Titanium (Life Sciences, Conn, USA) according to the manufacturer’s instructions. Sequences generated from 16S rRNA pyrosequencing were filtered, denoised, and analyzed using QIIME v1.9.1 and further clustered into OTUs based on 97% sequence similarity [20]. The barcodes in mapping dataset contained 351,590 reads (average number of reads per sample: 33,829; range: 3093 to 17579). From 16S rRNA gene sequencing results, we used PICRUSt to infer gene presentation using taxonomic information from Kyoto Encyclopedia of Genes and Genomes (KEGG) database [21, 22].

### Statistical analyses

All results are expressed as mean ± standard deviation (SD). Continuous variables were compared by independent t-test. Statistical calculations were performed using SPSS (SPSS Inc. Released 2007. SPSS for Windows, Version 16.0. Chicago, SPSS Inc.).

## Results

### Impact of co-administration of metformin and SB on serum total cholesterol, glucose and insulin tolerances, and other clinical parameters

**Fig 2:**
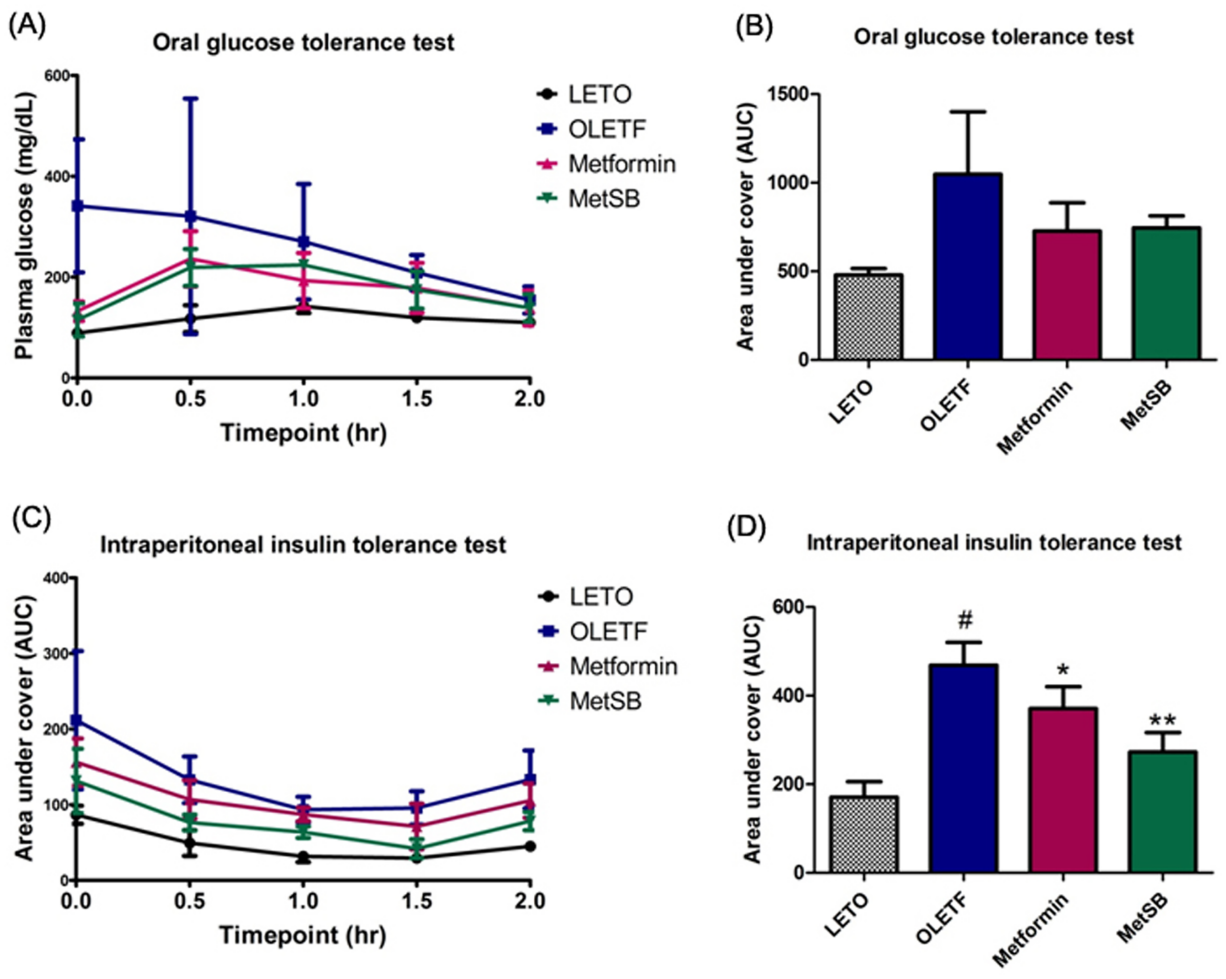
Effect of metformin and metformin plus *Scutellaria baicalensis* (SB) extract on the glucose homeostasis and insulin sensitivity in OLETF rats. (A, B) Oral glucose tolerance test (OGTT) and (C, D) intraperitoneal insulin tolerance test (IPITT). #: *p* < 0.05 compared to LETO group. *: *p* <0.05 compared to OLETF group. **: *p* <0.001 compared to OLETF group. MetSB: Metformin co-administered with *Scutellaria baicalensis* extract.

The level of serum total cholesterol in rats exposed to metformin and SB combination (MetSB group), but not metformin alone, was significantly lower compared to the OLETF group (Table 1, S1 dataset). However, the body weight or food intake of these two groups did not differ significantly from OLETF group that was devoid of any drug treatment. Not surprisingly, fasting glucose significantly decreased in both metformin group and MetSB group. To determine insulin sensitivity and ability to clear away blood glucose, OGTT and IPITT were conducted before the sacrifice (Fig 2). In OGTT, the AUC of both metformin and MetSB groups was significantly lower compared to OLETF group although there was no significant difference in this parameter between the former two groups. In IPITT, the AUC of both metformin and MetSB groups was significantly lower than OLETF group and the difference between MetSB and OLETF groups was more pronounced than that between MetSB and OLETF groups (p = 0.034 and p = 0.001, respectively).

**Table 1:** Changes of body weight, food intake and blood chemistry.

Parameters	LETO	OLETF	*P*^a^	Met^c^	*P*^2^^b^	MetSB^d^	*P*^2^ value
	(n = 7)	(n = 6)		(n = 7)		(n = 7)	
**Body weight**	499.6	612.9	<.001	598.9	.457	605.5	.649
**(g)**	(45.5)	(3.7)		(46.1)		(37.3)	
**Food intake**	81.3	54.1	<.001	60.8	.459	56.8	.596
**(g)**	(6.3)	(8.7)		(23.5)		(10.4)	
**Fasting glucose**	89.3	341.3	.010	132.5	.009	115.0	.004
**(mg/dL)**	(5.5)	(46.7)		(19.3)		(32.6)	
**Serum insulin**	0.25	0.52	.016	0.23	.013	0.25	.016
**(ng/mL)**	(0.03)	(0.19)		(0.03)		(0.03)	
**Triglyceride**	50.0	119.8	.007	138.4	.461	151.4	.104
**(mg/dL)**	(46.0)	(39.3)		(54.7)		(27.5)	
**Total cholesterol**	122	169.9	<.001	150.6	.264	143.7	.043
**(mg/dL)**	(15.1)	(21.2)		(40.4)		(24.1)	

Data are given as mean (standard deviation).

^a^P value: OLETF group compared to LETO group

^b^P^2^ value: Drug intervention groups compared to OLETF group

^c^Met: Metformin group

^d^MetSB: Metformin co-administered with *Scutellaria baicalensis* extract.

### Administration of SB up-regulated gene expressions of hepatic CYP7A1 and NR1H4

**Fig 3:**
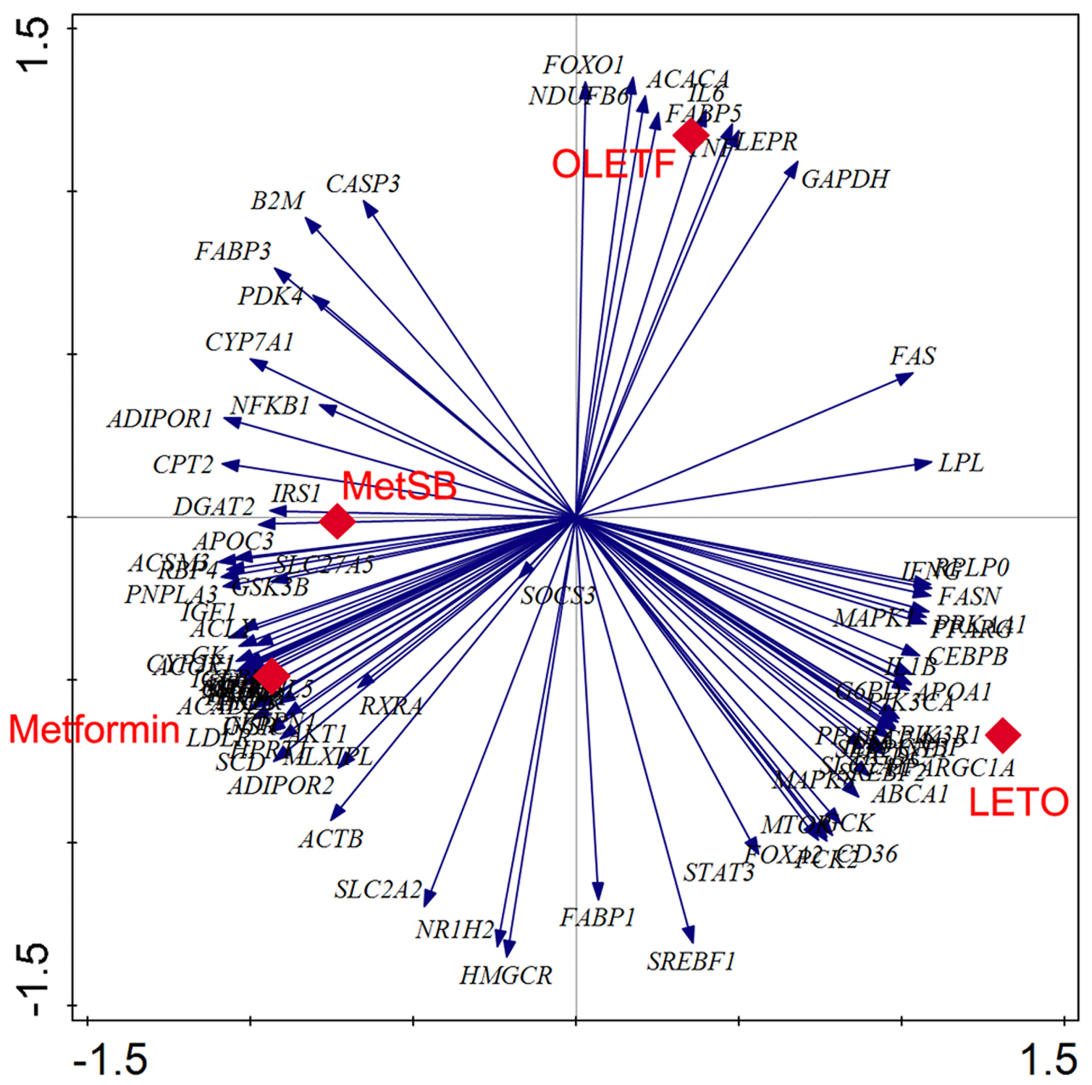
Principal Component Analysis (PCA) analyses of 84 gene sets of related to insulin resistance and fatty liver. Vectors indicate the strength and direction of each gene expressions to the overall distribution. Red plots correspond to each experimental groups in this study.

To search for the clues of action mechanism when SB is concurrently administered with metformin, we analyzed RNA extracted from the liver tissues using PCR array (Fig 3, S2 dataset). Among 85 key genes related to fatty liver and hepatic insulin resistance, six genes related to bile acid excretion were shown in Table 2. Among them, the expression of CYP7A1, LDLR, NR1H4 and HMGCR, the major genes related to bile acid synthesis were changed after combination treatment with metformin and SB. For instance, expression CYP7A1 was higher in both metformin and MetSB groups compared to OLETF group, but the difference was more pronounced in MetSB group (1.73-fold higher than metformin group). While, the expression of NR1H4 gene in OLETF rats was increased by 21.82- and 23.92-folds in response to the treatment with metformin and MetSB, respectively. The expression of LDLR and HMGCR genes were higher in both metformin and MetSB groups compared to OLETF group.

**Table 2:** Genes related to bile acid secretion from RT^2^ Profiler PCR array analysis.

Selected genes	Fold Changes (compared to LETO group)		
	OLETF	Metformin	MetSB
**CYP7A1**	20.393	26.028	44.942
**HMGCR**	0.429	1.008	0.940
**LDLR**	0.063	12.658	9.448
**NR1H4(FXR)**	1.064	22.816	23.918
**RXRA**	1.028	2.349	1.014
**SLC27A5**	8.815	44.694	83.286

No statistical analysis was available as we used pooled cDNA from four randomly selected rats from each group. MetSB: Metformin co-administered with *Scutellaria baicalensis* extract

### SB increases hepatic FXR and CYP7A1 expression

**Fig 4:**
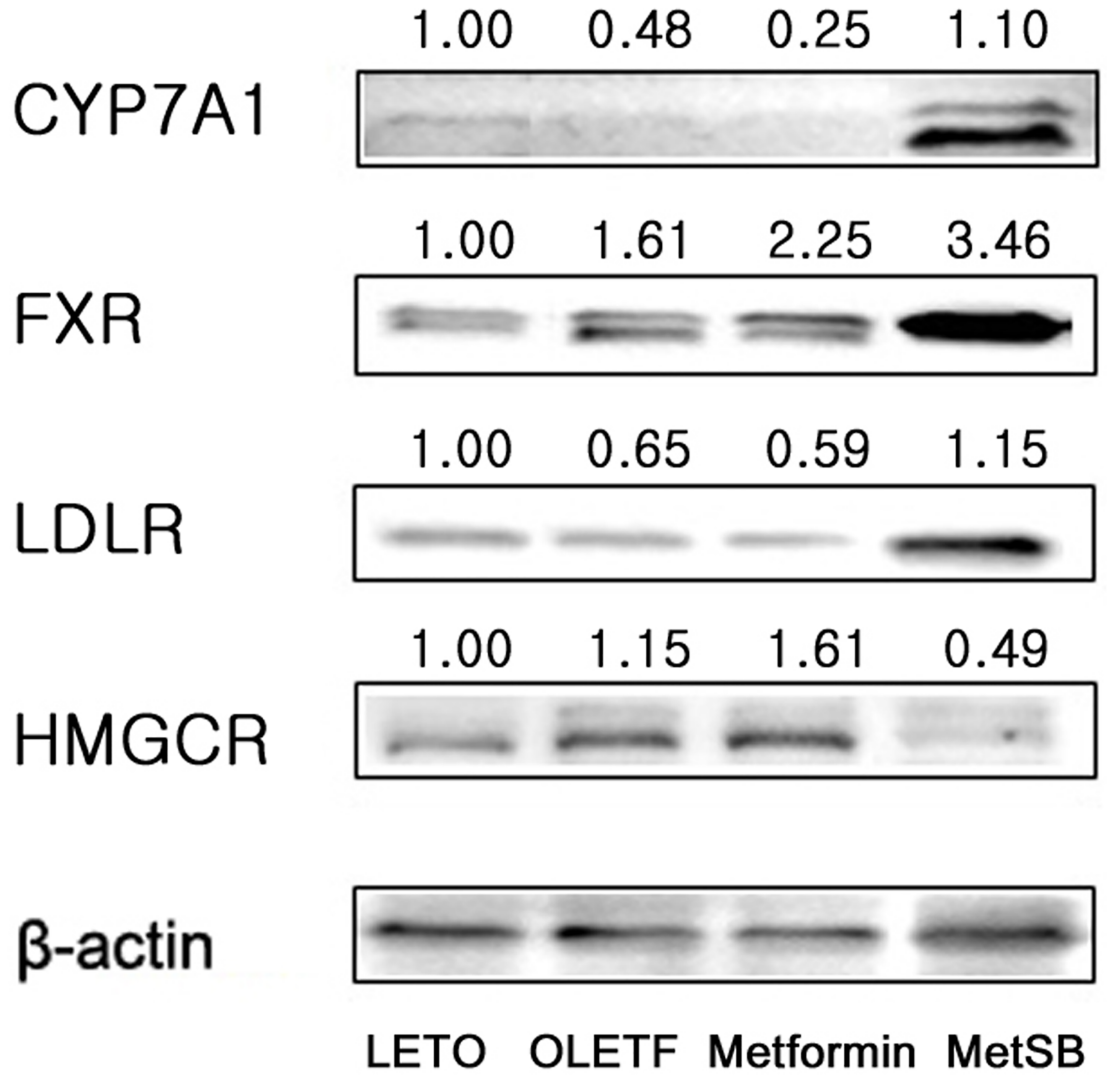
Western blot analysis of CYP7A1, HMG CoA reductase, LDL receptor and FXR in liver tissues. MetSB: Metformin co-administered with *Scutellaria baicalensis* extract. Each protein samples are obtained by pooling liver tissues from each group. Numbers denotes fold changes compared to LETO group. Y-axis denotes fold changes compared to LETO group.

On the basis of PCR array results, we confirmed protein expressions of four selected genes by western blot analysis (Fig 4). Combination treatment of metformin and SB increased hepatic CYP7A1 and LDLR expression while the metformin group maintained a level similar to the placebo group. Expression of CYP7A1 increased 1.1 times compared to the LETO group while OLETF and metformin group changed 0.48- and 0.25-fold, respectively. LDLR of MetSB group was 1.15 times higher than the LETO group. The MetSB group showed 0.49 times down-regulated expression of HMGCOA compared to the LETO group while the OLETF and metformin groups were up-regulated compared to the LETO group. Increase of hepatic FXR (NR1H4) expression was 3.46 times as much in the MetSB group compared to the metformin group (2.25-fold change compared to the LETO group).

### SB increases fecal excretion of cholesterol and bile acids

**Fig 5:**
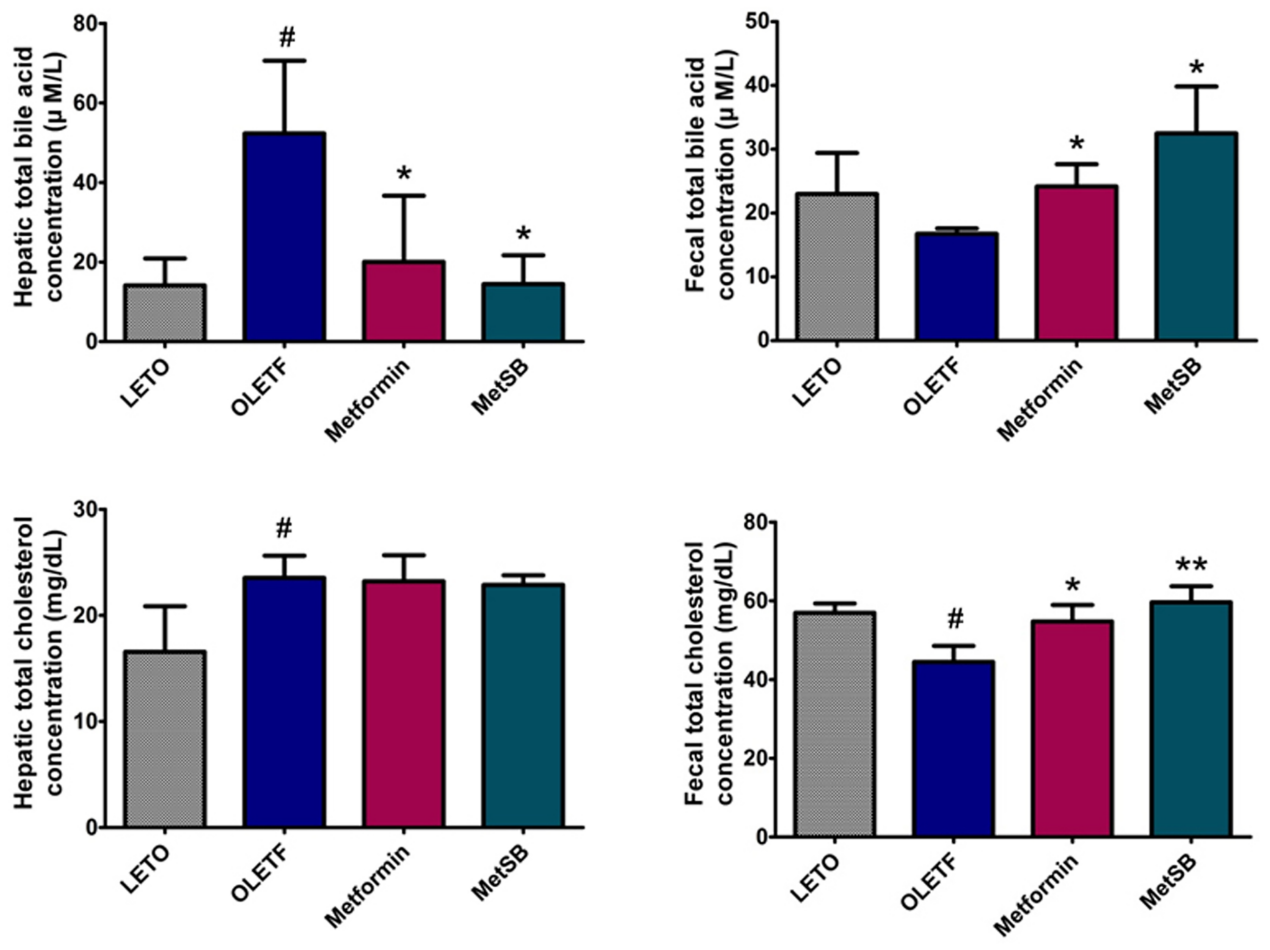
Quantitative analysis of total cholesterol and total bile acids in feces and liver. Values are means ± Standard deviations. #: *p* < 0.05 compared to LETO group. *: *p* <0.05 compared to OLETF group. **: *p* < 0.001 compared to OLETF group. MetSB: Metformin co-administered with *Scutellaria baicalensis* extract. Liver and fecal samples were obtained from individual rats from each group.

To investigate whether hepatic FXR and CYP7A1 expression changes have affected cholesterol and bile acid metabolism, we analyzed cholesterol concentration in liver tissue and bile acid concentration in feces (Fig 5, S3 dataset). Our results show that the hepatic cholesterol content of both the metformin and MetSB groups did not differ from placebo group. However, total cholesterol concentration significantly increased in the metformin group (p = 0.028) and MetSB group (p = 0.006). There was no difference between the metformin group and MetSB group statistically.

The total bile acid concentration in liver tissue significantly decreased in both the metformin and MetSB groups (p = 0.033 and p = 0.022, respectively) although there was no statistical difference between groups (p = 0.510). Fecal total bile acid concentration tended to increase, in contrast to liver tissue. Total bile acid increased in the metformin group (p = 0.021) and MetSB (p = 0.023), but there was no significant difference between these groups (p = 0.105).

### Shift of intestinal microbiota contributes to bile acid excretion

**Fig 6:**
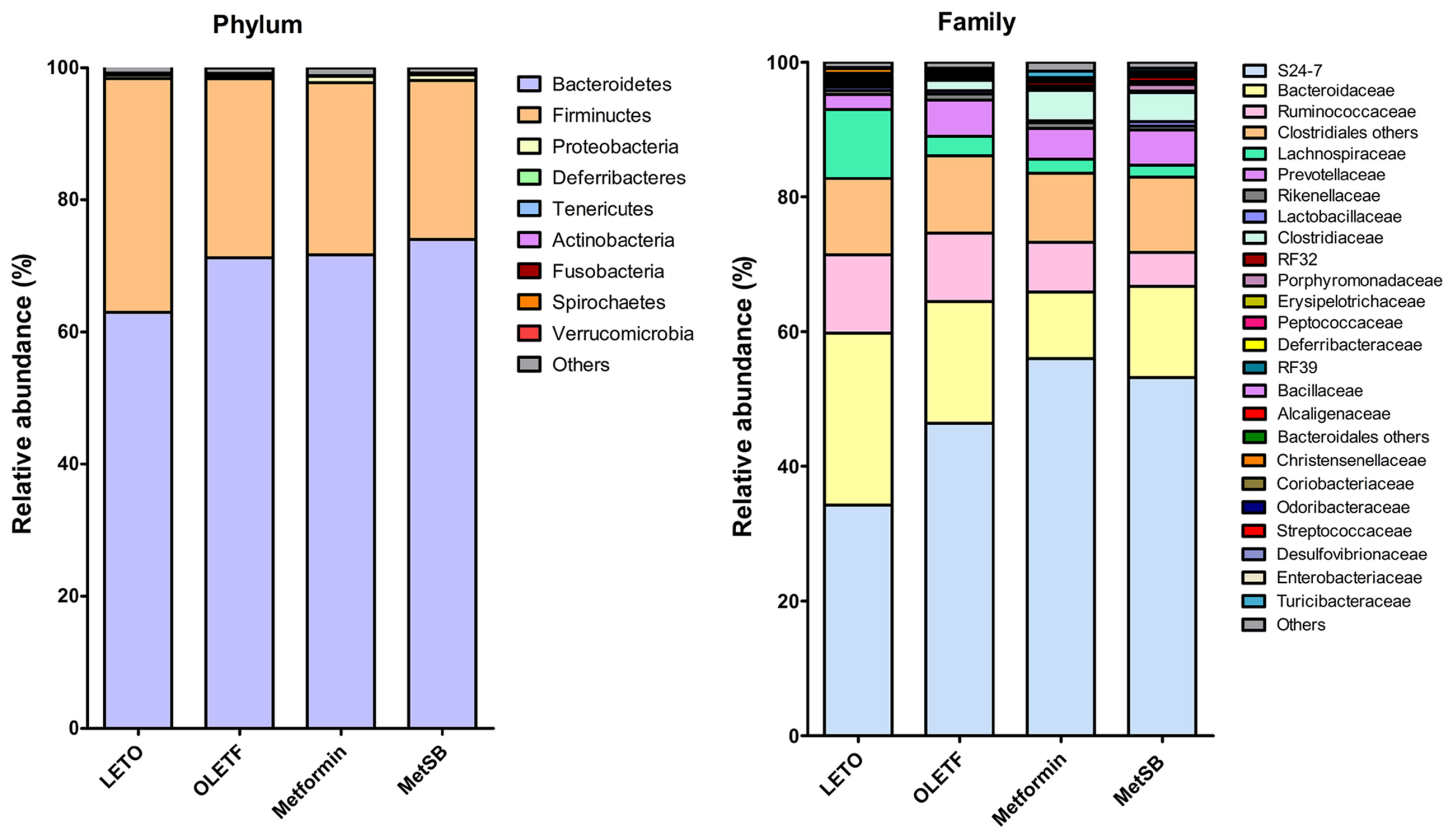
Relative abundance of intestinal microbial community after 12 weeks of drug intervention shown at the phylum level and family level. MetSB: Metformin co-administered with *Scutellaria baicalensis* extract. Data are from stool samples from 5 rats/group.

**Fig 7:**
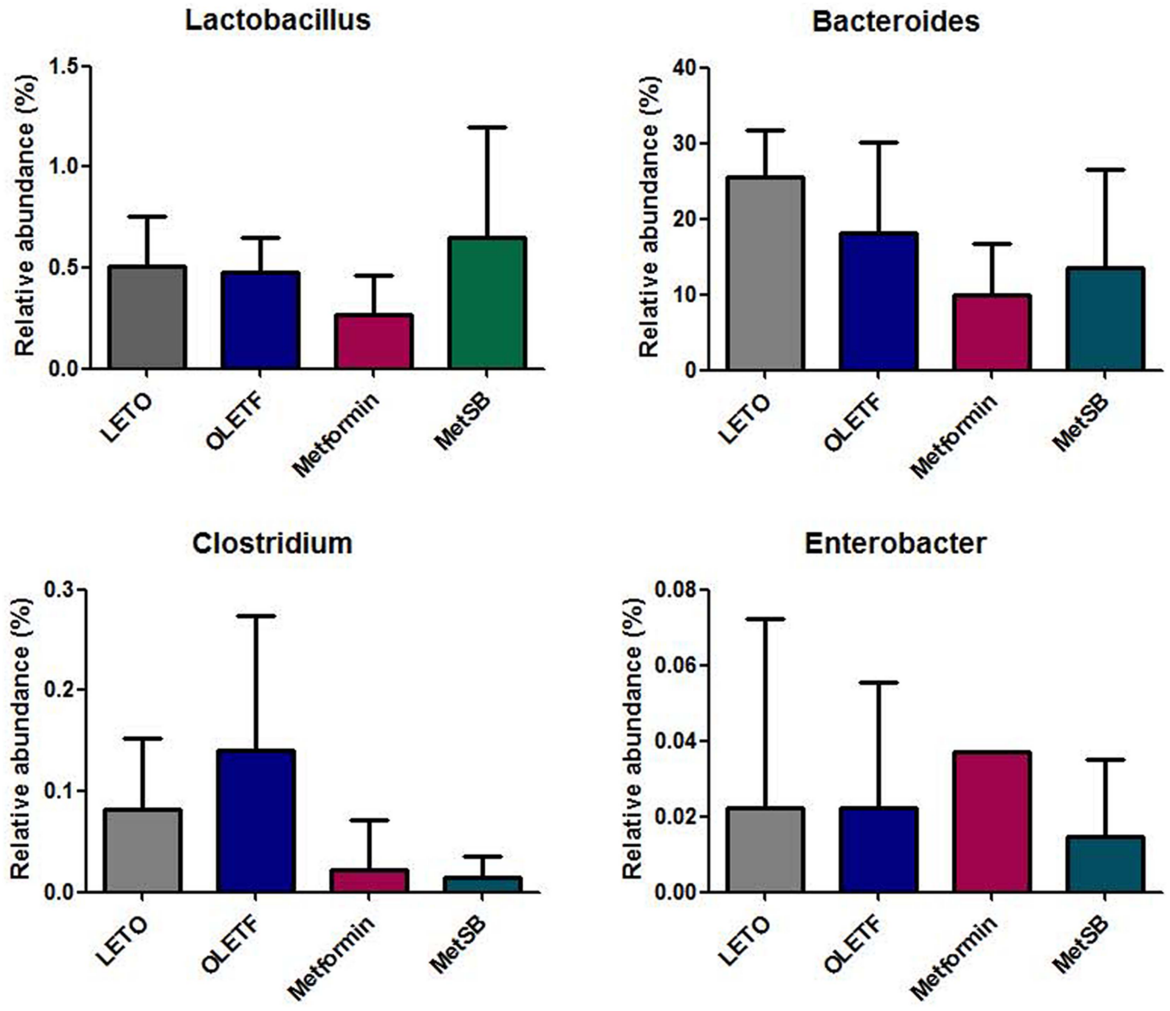
Relative abundance of intestinal microbiota with bile salt hydrolase (BSH) activity. Relative abundance of intestinal microbiota related to bile acid deconjugation was analyzed with fecal samples. There was no significant difference between groups. MetSB: Metformin co-administered with *Scutellaria baicalensis* extract. Data are from stool samples from 5 rats/group.

**Fig 8:**
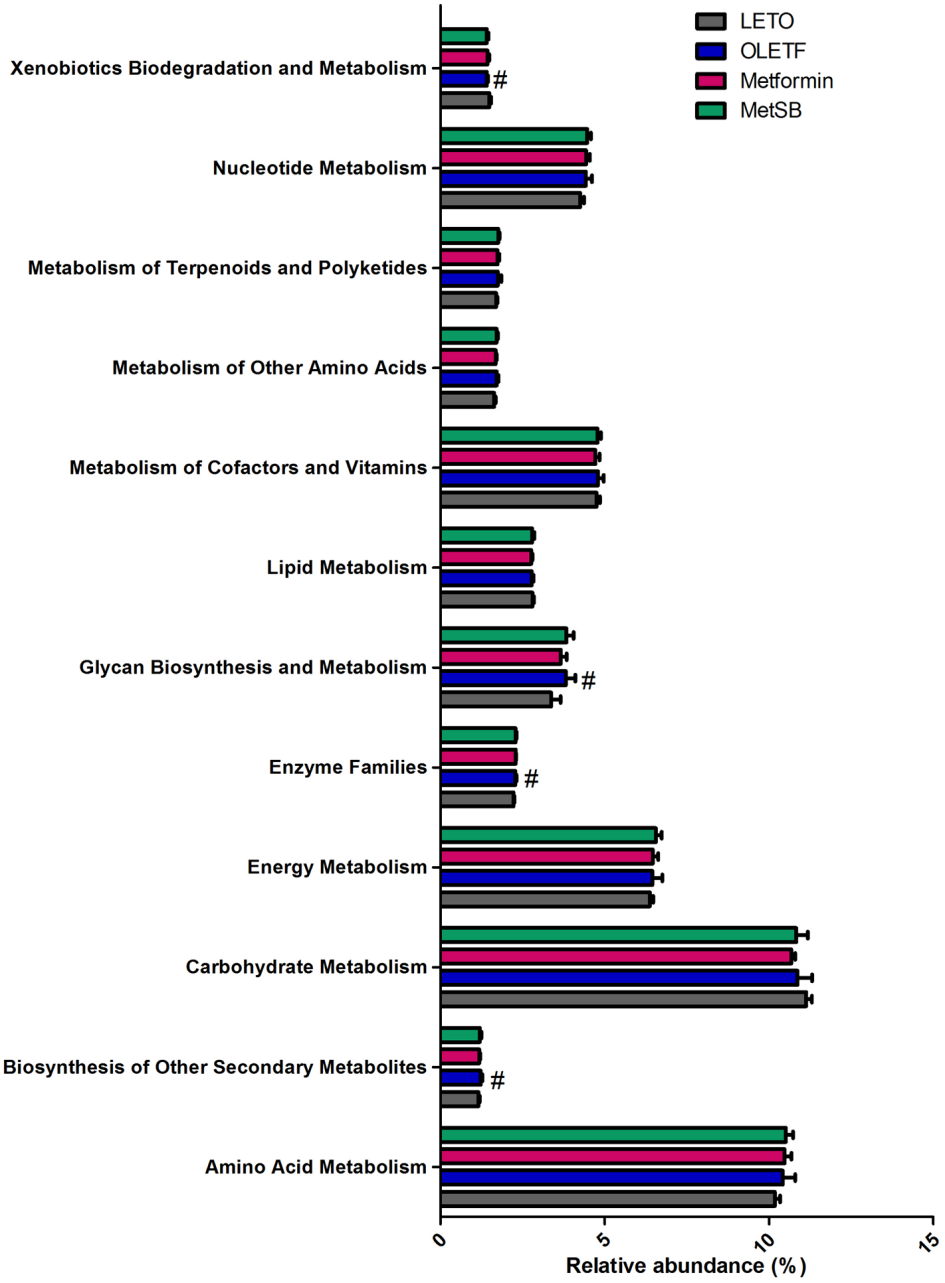
Functional composition of metagenome related to metabolism was predicted with PICRUSt algorithm. #: *p* < 0.05 compared to LETO group. MetSB: Metformin co-administered with *Scutellaria baicalensis* extract.

**Fig 9:**
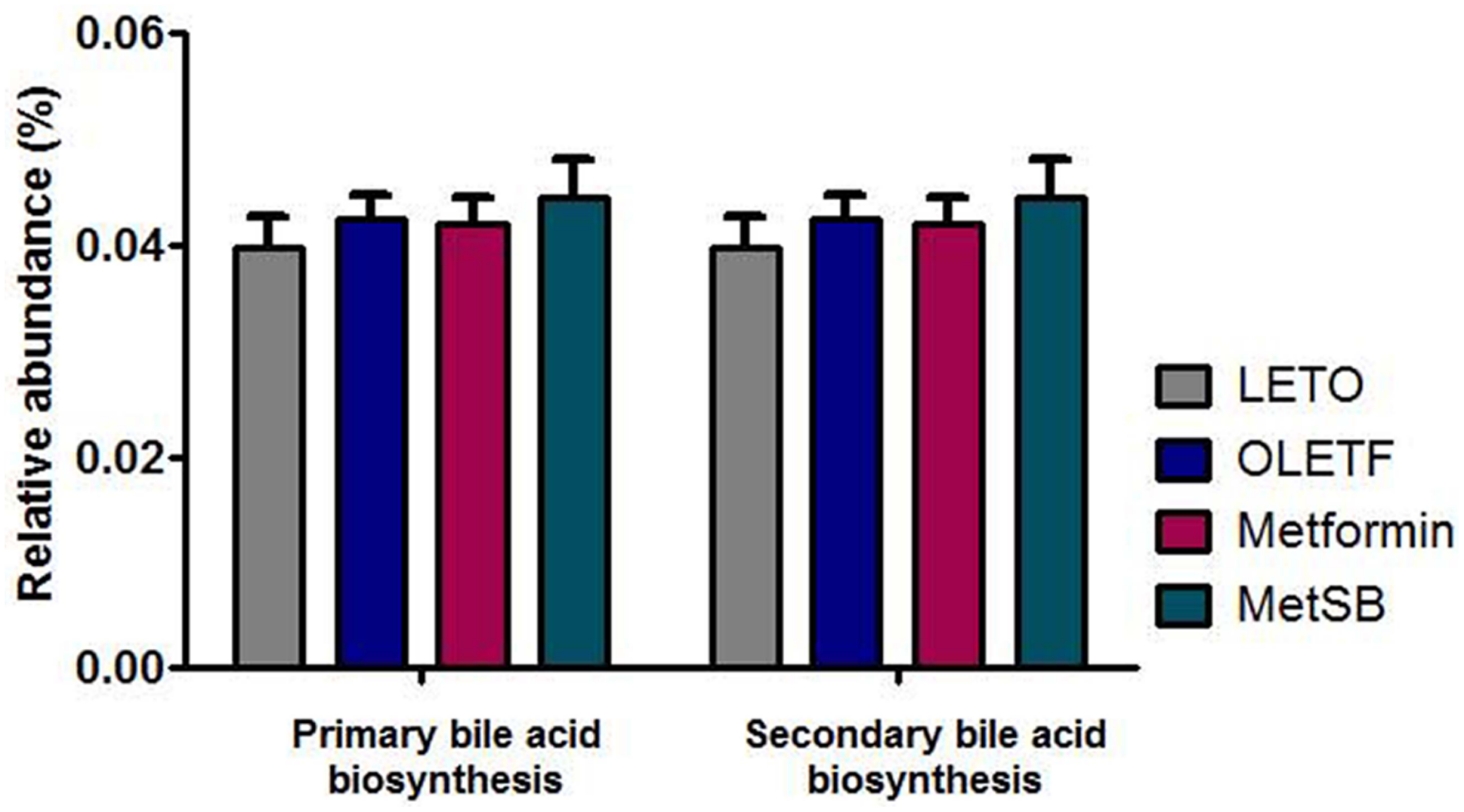
Predicted functional composition of metagenome related to primary and secondary bile acid biosynthesis was analyzed with PICRUSt algorithm. There was no significant difference between groups. MetSB: Metformin co-administered with *Scutellaria baicalensis* extract.

The taxanomical summary of each group by phylum and family level are in Fig 6 (S4 dataset). The most abundant phylum was *Bacteroidetes* (69.99 ± 4.87%) followed by *Firmicutes* (28.17 ± 4.99%). There were no significant differences between groups. We analyzed the relative abundance of several genera important in transformation of bile acids, such as *Bacteroides*, *Enterobacter*, *Clostridium*, *Bifidobacterium* and *Lactobacillus* (Fig 7). Although there was no statistical difference between groups, the relative composition of *Lactobacillus* and *Bacteroides* in MetSB group was higher than metformin group while *Clostridium* and *Enterobacter* were few. There was no *Bifidobacterium* found, unexpectedly. Since there was no statistical difference in representative BSH-active bacteria, we attempted to predict metagenomics functions related to metabolism by PICRUSt software package (Fig 8). Based on gene contents related to primary and secondary bile acid biosynthesis, predicted compositions of related microbiota are shown in Fig 9. Intestinal bacteria related to bile acid biosynthesis were increased in MetSB group compared to metformin group, although there was no statistical difference.

## Discussion

In this study, we found that to some degree, combination treatment is more effective in lowering glucose and serum cholesterol compared to metformin alone. There was an additional cholesterol lowering effect in the MetSB group but not in the metformin group. There is a continuous research on treating dyslipidemia with SB and its major components [23, 24]. In a study, treatment of Sprague-Dawley rats fed high fat diet with SB water extract for six weeks lowered triglyceride in plasma and liver significantly [25]. Another study administered SB to type 2 diabetic db/db mice for four weeks and showed marked improvement in insulin resistance and hypertriglyceridemia [26]. An experiment with hypercholesterolemic rabbits found that administration of SB lowers plasma total cholesterol and LDL-cholesterol [27]. This indicates that although metformin did not significantly lower cholesterol, a combination treatment with SB seems to produce an additional effect without interfering with the pharmaceutical efficacy of metformin.

To explore the action mechanisms, we screened for contributing genes with PCR array analysis. Using real-time polymerase chain reaction(PCR) assay with 84 gene sets of interest, the PCR array has the advantages of multi-gene profiling like microarray and reliable sensitivity [28]. We found that co-administration of metformin and SB affected a pathway involving FXR (farnesoid X receptor) and CYP7A1. FXR is a nuclear receptor encoded by NR1H4 gene. Bile acid activates FXR, which is mostly distributed in liver and intestine. FXR regulates hepatic bile acid content by repressing bile acid uptake and *de novo* synthesis while increasing bile acid secretion from the hepatocytes [29]. By modulation of FXR signaling, bile acid itself acts as a signaling molecule that affects glucose and lipid metabolism [13, 14]. Activation of FXR increases glycogen synthesis and decreases glycolysis [29]. Moreover, FXR protects beta-cell function, affecting glucose homeostasis [30]. Our result shows that the metformin group has both increased NR1H4 gene expression and hepatic FXR expression. The changes were maximized when SB is co-administered. Our result conflicts with a study which found that activation of AMPK by metformin can inhibit FXR transcriptional activity, perturb bile acid homeostasis, and injure the liver [31]. We assume that this is because our animal model differs from cholestasis model so that increased bile acid production and secretion did not induce toxicity. Another study with rat hepatocytes showed that metformin rather protects against glycochenodeoxycholic acid-induced apoptosis [32].

CYP7A1, also known as cholesterol 7-alpha-hydroxylase, is a rate-limiting enzyme important in cholesterol-to-bile acid conversion, and when bile acid increases in the liver, FXR suppresses CYP7A1 by negative feedback pathway [33]. Our study showed increased hepatic CYP7A1 in the MetSB group while the metformin group rather showed decreased expression of CYP7A1 compared to the control group. This suggests that although metformin and SB both act like FXR agonists, CYP7A1 expression was not inhibited when metformin is administered with SB. In western blot analysis, low-density lipoprotein receptor (LDLR) expression was increased in the MetSB group but not the metformin group, indicating circulating LDL-cholesterol is removed to the liver by endocytosis. This might have contributed to decreased serum cholesterol level in the metformin and MetSB group. LDL-cholesterol absorbed from the bloodstream is converted to biomolecules such as steroid hormones and cholesterol by the mevalonate pathway in the liver [34]. HMG-CoA reductase (HMGCR) catalyzes the HMG-CoA to mevalonic acid conversion, a key step of cholesterol biosynthesis [35, 36]. Interestingly, metformin and SB combination treatment, but not just metformin, lowered HMGCR level, suggesting a potential capability of lowering cholesterol by a mechanism similar to statin. However, total cholesterol in liver tissue remained the same, while fecal loss of cholesterol and bile acid increased. Although CYP7A1 expression increased in both the metformin and MetSB group, hepatic bile acid concentration significantly decreased. In sum, administration of metformin increased LDL-cholesterol absorption to the liver and activated a cholesterol-to-bile acid conversion by activation of CYP7A1. This process is promoted by metformin and SB combination treatment. By increased FXR activity, any excess cholesterol is discharged to the intestinal lumen by bile acid secretion and finally by feces. This also indicates bile acid reabsorption was decreased in this study.

One of the regulating mechanisms of bile acid reabsorption is mediated by intestinal microbiota. The role of intestinal microbiota in producing secondary bile acid in the small intestine has been known for years [37]. By shifting bile acid composition, intestinal microbiota affects host metabolism, and, conversely, bile acid composition can affect intestinal microbiota [38]. Some probiotic bacteria with BSH activity, mostly *Lactobacillus*, *Bifidobacterium*, *Clostridium* and *Bacteroides*, promote de-conjugation of bile acids that eventually fail to be reabsorbed and are excreted from the body through feces [33, 39]. Although our result did not show significant difference between groups, composition of *Lactobacillus* and *Bacteroides* were somewhat higher in the combination treatment group compared to the metformin group, while the opposite was the case in *Clostridium* and *Enterobacter*. However, these failed to explain loss of bile acids through feces. Thus, we used 16S rRNA gene contents to predict the functional composition of metagenome by PICRUSt algorithm. The result showed increased gene sets related to primary and secondary bile acid biosynthesis in metformin and SB combination treatment. This implies that intestinal microbiota might have contributed to lowering cholesterol level by cholesterol to bile acid conversion and de-conjugation of bile acids in MetSB group. Modulation of mouse intestinal microbiota by administration of probiotics for 21 days leads to increased CYP7A1 expression and augmented excretion of fecal bile acid [40]. This is in alignment with our result of increased CYP7A1 gene and protein expression after co-administration of metformin and SB. Nevertheless, the temporal sequence between microbiota and gene expression is still unknown. Conversely, increased secretion of bile acid in the intestinal tract favor Gram-positive bacteria such as *Lactobacillus* that cause dehydroxylation of primary bile acids to secondary bile acids, eventually leading to loss of bile acid through feces [38, 41]. Growing evidence indicates intestinal microbiota can also affect FXR activity [15, 42]. A comparison of FXR-deficient germ-free mice and wild-type mice showed that intestinal microbiota can regulate FXR signaling and bile acid synthesis [15]. Further study is needed to explain the contribution of intestinal bacteria to FXR activity and bile acid excretion in metformin and SB combination therapy.

## Supporting information

S1 Dataset Datasets of body weight changes and blood analysis. (SAV)

S2 Dataset Fold changes of 84 key genes related to fatty liver and hepatic insulin resistance. (XLS)

S3 Dataset Results of bile acid concentration in liver tissue and feces. (XLSX)

S4 Dataset Whole dataset of bacterial taxonomy. (XLSX)

S1 Checklist NC3Rs ARRIVE (Animal Research: Reporting In Vivo Experiments) Guidelines. (PDF)

## References

[1] Wulffelé eM, Kooy A, Zeeuw Dd, Stehouwer C, Gansevoort R. The effect of metformin on blood pressure, plasma cholesterol and triglycerides in type 2 diabetes mellitus: a systematic review. J Intern Med. 2004;256(1):1–14. doi: 10.1111/j.1365-2796.2004.01328.x 15189360 10.1111/j.1365-2796.2004.01328.x

[2] Huang W-H, Lee A-R, Yang C-H. Antioxidative and anti-inflammatory activities of polyhydroxyflavonoids of Scutellaria baicalensis georgi. Biosci Biotech Bioch. 2006;70(10):2371–80. 10.1271/bbb.50698 17031041

[3] Ye F, Xui L, Yi J, Zhang W, Zhang DY. Anticancer activity of Scutellaria baicalensis and its potential mechanism. J Altern Complem Med. 2002;8(5):567–72. 10.1089/107555302320825075 12470437

[4] Kim EH, Shim B, Kang S, Jeong G, Lee J-s, Yu Y-B, et al Anti-inflammatory effects of Scutellaria baicalensis extract via suppression of immune modulators and MAP kinase signaling molecules. J Ethnopharmacol. 2009;126(2):320–31. doi: 10.1016/j.jep.2009.08.027 19699788 10.1016/j.jep.2009.08.027

[5] Jung H-S, Kim MH, Gwak N-G, Im Y-S, Lee K-Y, Sohn Y, et al Antiallergic effects of Scutellaria baicalensis on inflammation in vivo and in vitro. J Ethnopharmacol. 2012;141(1):345–9. doi: 10.1016/j.jep.2012.02.044 22414480 10.1016/j.jep.2012.02.044

[6] Nagai T, Moriguchi R, Suzuki Y, Tomimori T, Yamada H. Mode of action of the anti-influenza virus activity of plant flavonoid, 5, 7, 4′-trihydroxy-8-methoxyflavone, from the roots of Scutellaria baicalensis. Antivir Res. 1995;26(1):11–25. 7741518 10.1016/0166-3542(94)00062-d

[7] Heo HJ, Kim D-O, Choi SJ, Shin DH, Lee CY. Potent inhibitory effect of flavonoids in Scutellaria baicalensis on amyloid β protein-induced neurotoxicity. J Agr Food Chem. 2004;52(13):4128–32. 15212458 10.1021/jf049953x

[8] Waisundara VY, Hsu A, Huang D, Tan BK-H. Scutellaria baicalensis enhances the anti-diabetic activity of metformin in streptozotocin-induced diabetic Wistar rats. Am J Chinese Med. 2008;36(03):517–40. 10.1142/S0192415X08005953 18543386

[9] Li H-B, Chen F. Isolation and purification of baicalein, wogonin and oroxylin A from the medicinal plant Scutellaria baicalensis by high-speed counter-current chromatography. J Chromatogr A. 2005;1074(1):107–10. 15941045 10.1016/j.chroma.2005.03.088

[10] Kovács G, Kuzovkina I, Szoke E, Kursinszki L. HPLC determination of flavonoids in hairy-root cultures of Scutellaria baicalensis Georgi. Chromatographia. 2004;60(1):S81–S5.

[11] Huang W-H, Chien P-Y, Yang C-H, Lee A-R. Novel synthesis of flavonoids of Scutellaria baicalensis Georgi. Chem Pharm Bull. 2003;51(3):339–40. 12612426 10.1248/cpb.51.339

[12] Horvath CR, Martos PA, Saxena PK. Identification and quantification of eight flavones in root and shoot tissues of the medicinal plant Huang-qin (Scutellaria baicalensis Georgi) using high-performance liquid chromatography with diode array and mass spectrometric detection. J Chromatogr A. 2005;1062(2):199–207. 15679157 10.1016/j.chroma.2004.11.030

[13] Tremaroli V, Bäckhed F. Functional interactions between the gut microbiota and host metabolism. Nature. 2012;489(7415):242–9. doi: 10.1038/nature11552 22972297 10.1038/nature11552

[14] Fiorucci S, Distrutti E. Bile Acid-Activated Receptors, Intestinal Microbiota, and the Treatment of Metabolic Disorders. Trends Mol Med. 2015;21(11):702–14. doi: 10.1016/j.molmed.2015.09.001 26481828 10.1016/j.molmed.2015.09.001

[15] Sayin SI, Wahlström A, Felin J, Jäntti S, Marschall H-U, Bamberg K, et al Gut microbiota regulates bile acid metabolism by reducing the levels of tauro-beta-muricholic acid, a naturally occurring FXR antagonist. Cell Metab. 2013;17(2):225–35. doi: 10.1016/j.cmet.2013.01.003 23395169 10.1016/j.cmet.2013.01.003

[16] Wilson I, Nicholson J. The role of gut microbiota in drug response. Curr Pharm Design. 2009;15(13):1519–23. 10.2174/138161209788168173 19442168

[17] Napolitano A, Miller S, Nicholls AW, Baker D, Van Horn S, Thomas E, et al Novel gut-based pharmacology of metformin in patients with type 2 diabetes mellitus. Plos One. 2014;9(7):e100778 doi: 10.1371/journal.pone.0100778 24988476 10.1371/journal.pone.0100778 PMC4079657

[18] Forslund K, Hildebrand F, Nielsen T, Falony G, Le Chatelier E, Sunagawa S, et al Disentangling type 2 diabetes and metformin treatment signatures in the human gut microbiota. Nature. 2015;528(7581):262–6. doi: 10.1038/nature15766 26633628 10.1038/nature15766 PMC4681099

[19] Food, Administration D. Guidance for industry: estimating the maximum safe starting dose in initial clinical trials for therapeutics in adult healthy volunteers. Center for Drug Evaluation and Research (CDER). 2005.

[20] Caporaso JG, Kuczynski J, Stombaugh J, Bittinger K, Bushman FD, Costello EK, et al QIIME allows analysis of high-throughput community sequencing data. Nat Methods. 2010;7(5):335–6. doi: 10.1038/nmeth.f.303 20383131 10.1038/nmeth.f.303 PMC3156573

[21] Kanehisa M, Goto S. KEGG: kyoto encyclopedia of genes and genomes. Nucleic Acids Res. 2000;28(1):27–30. 10592173 10.1093/nar/28.1.27 PMC102409

[22] Langille MG, Zaneveld J, Caporaso JG, McDonald D, Knights D, Reyes JA, et al Predictive functional profiling of microbial communities using 16S rRNA marker gene sequences. Nat Biotechnol. 2013;31(9):814–21. doi: 10.1038/nbt.2676 23975157 10.1038/nbt.2676 PMC3819121

[23] Seo M-J, Choi H-S, Jeon H-J, Woo M-S, Lee B-Y. Baicalein inhibits lipid accumulation by regulating early adipogenesis and m-TOR signaling. Food Chem Toxicol. 2014;67:57–64. doi: 10.1016/j.fct.2014.02.009 24560969 10.1016/j.fct.2014.02.009

[24] Pu P, Wang X-A, Salim M, Zhu L-H, Wang L, Xiao J-F, et al Baicalein, a natural product, selectively activating AMPKα 2 and ameliorates metabolic disorder in diet-induced mice. Mol Cell Endocrinol. 2012;362(1):128–38. 22698522 10.1016/j.mce.2012.06.002

[25] Yoon H-J, Park Y-S. Effect of Scutellaria baicalensis water extract on lipid metabolism and antioxidant defense system in rats fed high fat diet. J Korean Soc Food Sci Nutr. 2010;39(2):219–26.

[26] Song KH, Lee SH, Kim BY, Park AY, Kim JY. Extracts of Scutellaria baicalensis reduced body weight and blood triglyceride in db/db Mice. Phytother Res. 2013;27(2):244–50. doi: 10.1002/ptr.4691 22532505 10.1002/ptr.4691

[27] Króliczewska B, Miśta D, Zawadzki W, Wypchło A, Króliczewski J. Effects of a skullcap root supplement on haematology, serum parameters and antioxidant enzymes in rabbits on a high‐cholesterol diet. J Anim Physiol An N. 2011;95(1):114–24. 10.1111/j.1439-0396.2010.01033.x 20666864

[28] Chen Y, Gelfond JA, McManus LM, Shireman PK. Reproducibility of quantitative RT-PCR array in miRNA expression profiling and comparison with microarray analysis. BMC Genomics. 2009;10(1):407. 19715577 10.1186/1471-2164-10-407 PMC2753550

[29] Mazuy C, Helleboid A, Staels B, Lefebvre P. Nuclear bile acid signaling through the farnesoid X receptor. Cell Mol Life Sci. 2015;72(9):1631–50. doi: 10.1007/s00018-014-1805-y 25511198 10.1007/s00018-014-1805-y PMC11113650

[30] Popescu IR, Helleboid-Chapman A, Lucas A, Vandewalle B, Dumont J, Bouchaert E, et al The nuclear receptor FXR is expressed in pancreatic β-cells and protects human islets from lipotoxicity. FEBS Lett. 2010;584(13):2845–51. doi: 10.1016/j.febslet.2010.04.068 20447400 10.1016/j.febslet.2010.04.068

[31] Lien F, Berthier A, Bouchaert E, Gheeraert C, Alexandre J, Porez G, et al Metformin interferes with bile acid homeostasis through AMPK-FXR crosstalk. J Clin Invest. 2014;124(3):1037 doi: 10.1172/JCI68815 24531544 10.1172/JCI68815 PMC3938262

[32] Woudenberg-Vrenken TE, de la Rosa LC, Buist-Homan M, Faber KN, Moshage H. Metformin protects rat hepatocytes against bile acid-induced apoptosis. Plos One. 2013;8(8):e71773 doi: 10.1371/journal.pone.0071773 23951244 10.1371/journal.pone.0071773 PMC3741108

[33] Chiang JY. Bile acids: regulation of synthesis. J Lipid Res. 2009;50(10):1955–66. doi: 10.1194/jlr.R900010-JLR200 19346330 10.1194/jlr.R900010-JLR200 PMC2739756

[34] Brown M, Goldstein J. Lipoprotein receptors in the liver. Control signals for plasma cholesterol traffic. J Clin Invest. 1983;72(3):743 doi: 10.1172/JCI111044 6309907 10.1172/JCI111044 PMC1129238

[35] Istvan ES, Deisenhofer J. Structural mechanism for statin inhibition of HMG-CoA reductase. Science. 2001;292(5519):1160–4. doi: 10.1126/science.1059344 11349148 10.1126/science.1059344

[36] Buhaescu I, Izzedine H. Mevalonate pathway: a review of clinical and therapeutical implications. Clin Biochem. 2007;40(9):575–84. 17467679 10.1016/j.clinbiochem.2007.03.016

[37] Ridlon JM, Kang D-J, Hylemon PB. Bile salt biotransformations by human intestinal bacteria. J Lipid Res. 2006;47(2):241–59. doi: 10.1194/jlr.R500013-JLR200 16299351 10.1194/jlr.R500013-JLR200

[38] Islam KS, Fukiya S, Hagio M, Fujii N, Ishizuka S, Ooka T, et al Bile acid is a host factor that regulates the composition of the cecal microbiota in rats. Gastroenterology. 2011;141(5):1773–81. doi: 10.1053/j.gastro.2011.07.046 21839040 10.1053/j.gastro.2011.07.046

[39] Li G. Intestinal probiotics: interactions with bile salts and reduction of cholesterol. Procedia Environ Sci. 2012;12:1180–6.

[40] Degirolamo C, Rainaldi S, Bovenga F, Murzilli S, Moschetta A. Microbiota modification with probiotics induces hepatic bile acid synthesis via downregulation of the Fxr-Fgf15 axis in mice. Cell Rep. 2014;7(1):12–8. doi: 10.1016/j.celrep.2014.02.032 24656817 10.1016/j.celrep.2014.02.032

[41] Ridlon JM, Alves JM, Hylemon PB, Bajaj JS. Cirrhosis, bile acids and gut microbiota: unraveling a complex relationship. Gut microbes. 2013;4(5):382–7. doi: 10.4161/gmic.25723 23851335 10.4161/gmic.25723 PMC3839982

[42] Zhang X, Osaka T, Tsuneda S. Bacterial metabolites directly modulate farnesoid X receptor activity. Nutr Metab. 2015;12(1):1. 10.1186/s12986-015-0045-y PMC4657204 26604978

